# Positive correlation between post-cessation weight concerns and intentions to quit smoking in Chinese male smokers: A cross-sectional study

**DOI:** 10.18332/tid/200340

**Published:** 2025-02-21

**Authors:** Yunyun He, Chengzhang Diao, Lianyi Wen, Qing Zhou, Lilong Pang, Lingling Song, Yiwen Yang, Ting Chen

**Affiliations:** 1 Tongnan District Center for Disease Control and Prevention, Chongqing, People’s Republic of China; 2 Foreign Languages School Attached to Sichuan International Studies University, Chongqing, People’s Republic of China; 3 Public Health School, Chongqing Medical University, Chongqing, People’s Republic of China; 4 Shapingba District Center for Disease Control and Prevention, Chongqing, People’s Republic of China; 5 Chongqing Center for Disease Control and Prevention, Chongqing, People’s Republic of China

**Keywords:** post-cessation weight concerns, intentions to quit smoking, male smokers, post-cessation weight gain, perception

## Abstract

**INTRODUCTION:**

Concerns about post-cessation weight gain might influence the attempts to quit smoking. The knowledge about post-cessation weight gain, the post-cessation weight concerns, and their relationship to smoking quit intention have not been thoroughly studied in the Chinese population.

**METHODS:**

The present study is a cross-sectional study, which included a convenience sample of 1037 male smokers intending to quit smoking from Chongqing, China. Questionnaire-based investigations were conducted to assess sociodemographic characteristics, post-cessation weight concerns, and the intention to quit smoking. Multivariable logistic regression analysis was performed to assess the relationship between post-cessation weight concerns and intentions to quit smoking.

**RESULTS:**

The perception levels of post-cessation weight gain were low in the study population, and only 36.07% of respondents knew that ‘smoking cessation will affect the weight of smokers’. Generally, the medium post-cessation weight concern score was 2.16 on a 10-point scale, and only 8.29% of the participants had scores >5. Participants with a higher degree of concern regarding post-cessation weight gain had a higher adjusted odds ratio (AOR) for intentions to quit smoking (AOR=1.47; 95% CI: 1.09–1.97) compared to participants with lower weight concerns in the fully adjusted regression model.

**CONCLUSIONS:**

The extent of post-cessation weight concerns was low, partially due to the low perception of weight gain associated with smoking cessation. Moreover, the intentions to quit smoking were positively associated with post-cessation weight concerns. Consequently, it appears that weight concerns have not been a significant deterrent to the intention to quit smoking among the study population.

## INTRODUCTION

Tobacco smoking is an important cause of preventable premature mortality worldwide. In 2017, approximately 2.5 million people died from tobacco use^1^, and if current smoking rates continue, as many as 3 million people will die from tobacco use each year in China^2^. Smoking cessation can significantly reduce the occurrence of many chronic diseases and bring about immediate health benefits^3^, including decreased risks of stroke, cardiovascular disease, and smoking-related cancers^4-6^. Having an intention to quit is a prerequisite for preparing and taking smoking cessation action based on stage-based models of behavior change^7,8^. Intentions to quit smoking are associated with many factors, including gender, age, education level, race, level of nicotine dependence, self-efficacy, and past quit attempts^9^. However, the association between post-cessation weight concerns and intention to quit smoking was inconsistent or insufficient^10-12^.

Smoking cessation is associated with weight gain. One study revealed that approximately 80% of smokers would experience weight gain after quitting smoking^13^. A meta-analysis study found that the average weight gain was 4.1 kg in five years after smoking cessation, with 1.5 kg in continued smokers^14^. Some previous studies have shown that weight gain after quitting smoking may increase the risk of diabetes and hypertension, and weight gain is closely related to personal appearance^15-18^; it is proposed that fear of post-cessation weight gain might lower the intentions to attempt or seek treatment for quitting smoking and even reduce cessation rates^19^.

It is important to study the post-cessation weight concerns, particularly in people intending to quit smoking. The reported prevalence of weight concerns in smokers intending to quit smoking varies from 22.1% to 60.6%, depending on the study population and design^20-22^. Recent researchers have reported that higher levels of weight concerns were associated with woman^23^ experiencing weight gain in previous quit attempts^24^, higher BMI, and higher level cognition of post-cessation weight gain (for example, the cognition of ‘smoking cessation will affect the weight of smokers’)^25,26^ and more cigarettes per day^23^. As female smokers were more likely than male smokers to be concerned about post-cessation weight gain^27^, previous studies focused on concerns about weight gain after quitting among female smokers and the effects of related interventions^28,29^. Previous studies found that there are also post-cessation weight concerns among male smokers. Clark et al.^22^ reported that 25.17% of male smokers reported having weight concerns. In China, the majority of smokers are males^30^. However, the epidemiological characteristics of the perception of post-cessation weight gain and post-cessation weight concerns in male smokers have not been investigated until now. Therefore, the present study explores the characteristics about perception of weight gain associated with smoking cessation, and post-cessation weight concerns, and the associations between post-cessation weight concerns and intentions to quit smoking in male smokers from Chongqing, the largest metropolitan city in China.

## METHODS

### Study population

The present study is a cross-sectional study, which is consisted of 1037 male smokers planning to quit smoking from four districts of Chongqing: Yuzhong, Shapingba, Jiulongpo and Bishan . Convenience sampling was used and the participants were surveyed in parks or city squares selected from these districts in July 2017. Eligibility criteria of the study were residents who were males, aged 18–80 years, currently smoking at least 1 cigarette per day (including e-cigarettes), and intentions to quit smoking in the next year. Exclusion criteria included a diagnosis of current psychopathology, cancer, myocardial infarction, and severe stroke diseases. Participants voluntarily enrolled in the trial following informed consent procedures and completed a 9–13 minutes face-to-face questionnaire survey outdoors. A total of 1179 subjects were surveyed among 1215 eligible subjects (response rate 97.04%). Among the 1179 subjects, 1037 male smokers finished all questionnaires and were included. Study procedures were by institutional guidelines and approved by the Institutional Review Board of Chongqing Medical University.

The present investigation included interviewer administration of screening and baseline interviews. Screening interviews collected sociodemographic, smoking status, intentions to quit smoking, and disease history. Baseline interviews included questions relevant to the study aims (sociodemographic, perception of weight gain after smoking cessation, weight concerns, intentions to quit, smoking situation, and others). The questionnaire was designed based on literature and an expert consultation, and the validity of the questionnaire was tested through a small sample survey (n=33). For difficult questionnaire questions, the investigator explained them further so that the subject could better understand the question. Training procedures for Chongqing Medical University undergraduate interview staff included: 1) intensive training by the project leader, 2) assessment of participant comprehension of items, 3) unified verification standard to ensure data collection integrity, and 4) double data entry of participant responses to ensure accurate response coding.

### Measures

*Sociodemographic characteristics*

The present study included sociodemographic characteristics such as age (≤29, 30–40, 40–50, 50–60, ≥60 years), race (Han nationality, ethnic minorities), region (urban district, villages, and towns), marital status (single or married/living with a partner), an education level (low: illiterate or primary school; medium: high school or technical secondary school; and high: university or junior college), monthly income (in US$ equivalent) (<270, 270–680, ≥680). Self-reported height and weight were collected to calculate the BMI (kg/m^2^).

*Perception of weight gain after quitting smoking*

Participants were asked four items to assess their perception of weight gain after smoking cessation. Responses categories included: ‘agree’, ‘neither agree nor disagree’, ‘disagree’, and ‘don’t know’. Pre-survey results showed that the internal consistency of this item was high (α=0.879).

*Post-cessation weight concerns*

Participant weight concerns score was obtained by calculating the mean response to 6 items using a 10-point Likert scale (1=not at all, to 10=very much)^31^. Internal consistency was moderately high (α=0.802). A score ≥5 indicated clear concerns about weight gain after quitting smoking. For the analysis of associations between the perception of post-cessation weight gain and post-cessation weight concerns, and post-cessation weight concerns and intentions to quit smoking, post-cessation weight concerns score was dichotomized at the median (score ≤2.16 defined higher weight concerns, score ≤2.16 defined lower weight concerns).

*Intentions to quit smoking*

The intention to quit smoking score was measured using the question: ‘How strong is your intention to quit?’. The responses were scored on a 10-point scale that ranged from 1 (being not strong at all) to 10 (being extremely strong). For the analysis of associations between post-cessation weight concerns and intentions to quit smoking, the intentions to quit smoking score was dichotomized at the median (a score >5 defined higher intentions to quit smoking, and a score ≤5 defined lower intentions to quit smoking).

*Other smoking-related variables*

Nicotine dependence was measured by the Fagerström test for nicotine dependence^32^. Participant’s nicotine dependence score was obtained by calculating the sum of responses to 6 items (0=not at all, to 10=very much). This 6-item self-report measure has adequate internal consistency (α=0.879). The age started smoking was measured by asking: ‘How old where you when you started smoking regularly?’. The variable was converted into three ranges: <18, 18–22, and ≥23 years. Similarly, smoking duration was measured using the question: ‘How long have you been smoking?’. The variable was classified as ≤10 and >10 years. Self-reported cigarettes per day were measured by the question: ‘How many cigarettes do you smoke every day?’. The variable was converted into four ranges: 1–10, 11–20, 21–30, and ≥31. We assessed variables related to smoking cessation situation, including whether or not to attempt smoking cessation (no, yes), the total number of previous smoking cessation attempts (1–5, >5), whether or not gained weight in previous smoking cessation attempts (no, yes) and the maximum amount of weight gain in quitting smoking attempts (<5, ≥5 kg). Controlling weight gain efficacy after quitting (WEAQ) score was obtained by calculating the mean response to 6 items using a 10-point Likert scale (1=not at all confident, to 10=very confident)^31^. Internal consistency was moderately high (α= 0.855).

### Statistical analysis

We carried out descriptive analyses of participants’ characteristics, the perception of weight gain after quitting smoking, and post-cessation weight concerns. All categorical variables were described as frequencies and percentages, while continuous variables were summarized as median and interquartile range (IQR). Unadjusted (OR) and adjusted odds ratio (AOR) and 95% confidence intervals (95% CI) were calculated using multiple logistic regression analysis to explore the associations between the independent variable (post-cessation weight concerns) and dependent variable (intentions to quit smoking). The unadjusted odds ratio in the crude model did not control for any variables. Adjusted odds ratio in Model 1 was adjusted for age, race, region, marital status, education level, occupation, income, and BMI. Adjusted odds ratio in Model 2 was additionally adjusted for age of starting smoking, smoking duration, nicotine dependence score, cigarettes, smoking cessation attempts, and controlling weight efficacy after quitting smoking. All analyses were performed in SPSS 25.0. An α level of p<0.05 was used to determine the level of statistical significance.

## RESULTS

### Sample characteristics

Table 1 shows the sample characteristics of the male smokers investigated in the cross-sectional study. In the survey, participants consisted of primarily Han nationality (95.76%), urban district (67.21%), married or living with a partner (67.89%), medium or high level of education (86.69%), and medium or high income (76.55%). Most of the subjects had a high level of smoking duration (>10 years, 66.06%). Almost half of the sample started smoking at an age of 18–23 years, while the proportion of those that started smoking at an age of <18 years or >23 years was 39.73% and 13.69%, respectively. More than half of the smokers (67.02%) had attempted to quit smoking before, and almost one-fifth (20.43%) had gained weight in an attempt. The BMI of the majority of participants (60.2%) was normal. The nicotine dependence score (score=3) was below the cutoff, reflecting a low-level of dependence on nicotine (score=6); on the contrary, the controlling weight efficacy after quitting smoking score (score=7.67) was above the cutoff, reflecting high efficacy (score=6).

**Table 1:** Characteristics of the study population (N=1037)

*Characteristics*	*n*	*%*
**Age** (years)		
<30	340	32.79
30	218	21.02
40	197	19.00
50	176	16.97
≥60	106	10.22
**BMI** (kg/m^2^)		
<18.5	55	5.30
18.5–23.9	624	60.20
≥24	358	34.50
**Race**		
Han nationality	993	95.76
Ethnic minorities	44	4.24
**Region**		
Urban district	697	67.21
Villages and towns	340	32.79
**Marital status**		
Single	333	32.11
Married/living with partner	704	67.89
**Education level**		
Low	138	13.31
Medium	494	47.64
High	405	39.05
**Monthly income^a^** (in US$)		
<270	224	23.46
270–680	375	39.27
≥680	356	37.28
**Age started smoking** (years)		
<18	412	39.73
18–22	483	46.58
≥23	142	13.69
**Smoking duration** (years)		
≤10	352	33.94
>10	685	66.06
**Nicotine dependence score,** median (IQR)	3.00 (1.00–5.00)	
**Cigarettes/day**		
1–10	97	9.35
11–20	156	15.04
21–30	477	46.00
≥31	307	29.60
**Intentions to quit smoking score,** median (IQR)	5.00 (3.00–8.00)	
**Smoking cessation attempts**		
No	342	32.98
Yes	695	67.02
**Number of smoking cessation attempts^b^**		
1–5	621	89.35
>5	74	10.65
**Weight gain in smoking cessation attempts^b^**		
No	553	79.57
Yes	142	20.43
**Maximum amount of post-cessation weight gain^c^,** median (IQR)	5.00 (4.00–10.00)	
**Controlling WEAQ,** median (IQR)	7.67 (5.67–9.17)	

BMI: body mass index. WEAQ: weight efficacy after quitting smoking. IQR: interquartile range.

a Data were missing for 82 individuals.

b A total of 695 of the respondents had tried to quit smoking.

c A total of 142 people gained weight in smoking cessation attempts.

### The perceptions of post-cessation weight gain and post-cessation weight concerns

The perceptions of post-cessation weight gain are given in Table 2. Only 374 (36.07%) participants knew that ‘smoking cessation will affect the weight’. Less than one-quarter of the sample (22.37%) knew that ‘weight gain after smoking cessation belongs to withdrawal symptoms’. Those knowing ‘weight gain after smoking cessation will occur in some smokers’ and ‘weight gain will be rapid in the first three months after smoking cessation, then slow down and stabilize’ accounted for only 29.60% and 14.95%, respectively.

**Table 2:** The perception of weight gain after quitting smoking (N=1037)

*Variable*	*n*	*%*
**Smoking cessation will affect the weight of smokers**		
Agree	374	36.07
Neither agree nor disagree	151	14.56
Disagree	151	14.56
Don’t know	361	34.81
**Weight gain after smoking cessation belongs to withdrawal symptoms**		
Agree	232	22.37
Neither agree nor disagree	148	14.27
Disagree	87	8.39
Don’t know	570	54.97
**Weight gain after smoking cessation will occur in some smokers**		
Agree	307	29.60
Neither agree nor disagree	117	11.28
Disagree	78	7.52
Don’t know	535	51.59
**Weight gain will be rapid in the first three months after smoking cessation, then slow down and stabilize**		
Agree	155	14.95
Neither agree nor disagree	112	10.80
Disagree	61	5.88
Don’t know	709	68.37

The 6-item scale that measured concerns about post-cessation weight gain is given in Table 3. Around 14.95% of the subjects reported no weight concerns (1 on a 10-point scale). The median scores of item 1 and item 5 were 3 and 2, respectively, while those of item 2, item 3, item 4, and item 6 were just 1. Notably, the median sum score of weight concerns derived from the six items was 2.16 (1.33–3.33). And only 8.29% of the participants had high post-cessation weight concerns (score ≥5 on a 10-point scale).

**Table 3:** The score of post-cessation weight concerns scale (N=1037)

*Questions*	*Score* *Median (IQR)*	*Score ≥5* *n (%)*
1. How important is losing weight or maintaining your current weight compared with other personal health concerns?	3 (1–6)	448 (43.20)
2. People smoke for many reasons. Compared with all of your reasons for smoking, how important is smoking to control your weight?	1 (1–1)	56 (5.40)
3. How much do cigarettes help you to control your weight?	1 (1–2)	130 (12.54)
4. How concerned are you about gaining weight as a result of quitting?	1 (1–3)	168 (16.20)
5: How likely do you think it is that you will gain weight as a result of quitting?	2 (1–5)	301 (29.03)
6. How likely is it that you would go back to smoking after quitting if you gained too much weight?	1 (1–3)	202 (19.48)
Weight concerns score	2.16 (1.33–3.33)	86 (8.29)

IQR: interquartile range.

Table 4 displays the characteristics of subjects with different degrees of post-cessation weight concerns. Those with higher weight concerns (score ≤2.16) were younger, single, living in an urban district, had a high level of education, started smoking early, had shorter smoking duration but were heavy smokers, experienced weight gain in previous quit attempts, and knew more about post-cessation weight gain.

**Table 4:** Post-cessation weight concerns, lower degree versus higher degree (N=1037)

*Characteristics*	*Post-cessation weight concerns*		*χ^2^*	*p*
	*Lower degree* *(score ≤2.16)* *n (%)*	*Higher degree* *(score >2.16)* *n (%)*		
**Age** (years)			37.77	<0.01
<30	176 (27.76)	164 (40.69)		
30	132 (20.82)	86 (21.34)		
40	118 (18.61)	79 (19.60)		
50	120 (18.93)	56 (13.90)		
≥60	88 (13.88)	18 (4.47)		
**BMI** (kg/m^2^)			0.02	0.89
≤24	414 (65.30)	265 (65.76)		
>24.0	220 (34.70)	138 (34.24)		
**Race**			2.40	0.08
Han nationality	612 (96.53)	381 (94.54)		
Ethnic minorities	22 (3.47)	22 (5.46)		
**Region**			4.79	0.03
Urban district	410 (64.67)	287 (71.22)		
Villages and towns	224 (35.33)	116 (27.78)		
**Marital status**			23.58	<0.01
Single	168 (26.50)	165 (40.94)		
Married/living with partner	466 (73.50)	238 (59.06)		
**Education level**			24.67	<0.01
Low	263 (41.48)	119 (29.53)		
Medium	276 (43.54)	180 (44.66)		
High	95 (14.98)	104 (25.81)		
**Monthly income^a^** (in US$)			4.08	0.13
<270	131 (20.66)	93 (23.08)		
270–680	243 (38.33)	132 (32.75)		
≥680	260 (41.01)	149 (36.97)		
**Age started smoking** (years)			7.16	0.03
<18	249 (39.27)	163 (40.45)		
18–22	284 (44.80)	199 (49.38)		
≥23	101 (15.93)	41 (10.17)		
**Smoking duration** (years)			18.77	<0.01
≤10	183 (28.86)	169 (41.94)		
>10	451 (71.14)	234 (58.06)		
**Cigarettes/day**			15.54	<0.01
1–10	72 (11.35)	25 (6.95)		
11–20	97 (15.30)	59 (14.64)		
21–30	301 (47.48)	176 (43.67)		
≥31	164 (25.87)	143 (35.48)		
**Smoking cessation attempts**			4.65	0.04
No	225 (35.49)	117 (29.03)		
Yes	409 (64.51)	286 (70.97)		
**Weight gain in smoking cessation attempts^b^**			93.45	<0.01
No	376 (91.93)	177 (61.89)		
Yes	33 (8.07)	109 (38.11)		
**Perception of smoking cessation will affect the weight of smokers**			163.81	<0.01
Agree	138 (21.77)	236 (58.56)		
Neither agree nor disagree	360 (56.78)	152 (37.72)		
Disagree	136 (21.45)	15 (3.72)		

BMI: body mass index.

a Data were missing for 82 individuals.

b A total of 695 of the respondents had tried to quit smoking.

### Associations between post-cessation weight concerns and intentions to quit smoking

Table 5 shows that post-cessation weight concerns are positively association with intention to quit smoking in the study population. In the crude model, Model 1 and Model 2, those with higher weight concerns (score ≤2.16) had a higher odds ratio for intentions to quit smoking (crude model: OR=1.39; 95% CI: 1.09–1.18; Model 1: AOR=1.40; 95% CI: 1.07–1.83; Model 2: AOR=1.47; 95% CI: 1.09–1.97, respectively) compared to participants with lower weight concerns (score ≤2.16). In addition to weight concerns, the age of starting smoking, smoking cessation attempts, and controlling weight efficacy after quitting smoking were influencing factors for intentions to quit smoking in Model 2 (Table 6).

**Table 5:** Associations between post-cessation weight concerns and intentions to quit smoking (N=1037)

*Model*	*OR (95% CI)*	*AOR (95% CI)*	*p*
Crude model	1.39 (1.09–1.78)		0.01
Model 1		1.40 (1.07–1.83)	0.01
Model 2		1.47 (1.09–1.97)	0.01

Dependent variable: intentions to quit smoking. Independent variable: post-cessation weight concerns. Crude model: not controlled for any variables. AOR: adjusted odds ratio. Model 1: adjusted for age, race, region, marital status, education level, occupation, income, BMI. Model 2: adjusted as for Model 1 plus age of starting smoking, smoking duration, nicotine dependence score, cigarettes, smoking cessation attempts, controlling weight efficacy after quitting smoking.

**Table 6:** Logistic regression results of influencing factors for intentions to quit smoking (N=1037)

*Variables*	*Model 2* *AOR (95% CI)*	*p*
**Age** (years)		0.15
<30 ®	1	
30	1.27 (0.72–2.24)	0.41
40	1.63 (0.84–3.17)	0.15
50	2.25 (1.12–4.49)	0.02
≥60	1.88 (0.82–4.33)	0.14
**Race**		
Han nationality ®	1	
Ethnic minorities	1.64 (0.83–3.24)	0.15
**Region**		
Urban district ®	1	
Villages and towns	1.02 (0.76–1.37)	0.90
**Marital status**		
Single ®	1	
Married/living with partner	0.99 (0.63–1.55)	0.96
**Education level**		0.87
Low ®	1	
Medium	0.90 (0.56–1.44)	0.65
High	0.86 (0.49–1.52)	0.61
**Occupation**		0.17
Government agency and public institution ®	1	
Workers and business service	1.11 (0.76–1.61)	0.60
Farmer	1.12 (0.52–2.42)	0.77
Unemployed	1.23 (0.73–2.06)	0.44
Retiree	0.40 (0.18–0.88)	0.02
Other workers	0.99 (0.50–1.97)	0.98
**Monthly income^a^** (in US$)		0.69
<270 ®	1	
270–680	1.14 (0.77–1.69)	0.50
≥680	1.20 (0.79–1.81)	0.40
**BMI** (kg/m^2^)	0.96 (0.92–1.01)	0.11
**Age started smoking** (years)		0.08
<18 ®	1	
18–22	1.41 (1.03–1.93)	0.03
≥23	1.45 (0.89–2.35)	0.13
**Smoking duration** (years)		
≤10 ®	1	
>10	0.73 (0.44–1.20)	0.22
**Nicotine dependence score**	0.99 (0.91–1.07)	0.71
**Cigarettes/day**		0.38
1–10 ®	1	
11–20	1.21 (0.67–2.20)	0.53
21–30	1.13 (0.62–2.05)	0.69
≥31	1.58 (0.75–3.33)	0.23
**Smoking cessation attempts**		
No ®	1	
Yes	3.07 (2.24–4.19)	<0.01
**Controlling weight efficacy after quitting smoking**	1.12 (1.04–1.20)	<0.01
**Post-cessation weight concerns**		
Lower degree (score ≤2.16) ®	1	
Higher degree (score >2.16)	1.47 (1.09–1.97)	0.01

AOR: adjusted odds ratio. Model 2: adjusted for age, race, region, marital status, education level, occupation, income, BMI, age of starting smoking, smoking duration, nicotine dependence score, cigarettes, smoking cessation attempts, controlling weight efficacy after quitting smoking. ® Reference categories.

## DISCUSSION

We conducted the study to demonstrate the knowledge about post-cessation weight gain and post-cessation weight concerns in Chinese male smokers. The results showed that the levels of knowledge about post-cessation weight gain and post-cessation weight concerns were quite low in the population, and post-cessation weight concerns were positively associated with the intention to quit smoking in the study population.

The study found that 14.95% of the participants reported no weight concerns (1 on a 10-point scale), similar to other studies^33,34^. Nonetheless, only 8.29% of the participants had high weight concerns (≥5 on a 10-point scale) about quitting smoking, which was quite low compared to other study results, with over 50% concerns reported in female smokers and over 25% in male smokers of Western countries^20,22,35^. It may be related to the early development of health education on smoking cessation in Western countries. The perception level of post-cessation weight gain was higher in Western countries so the level of post-cessation weight concerns is higher. In contrast, the lower levels of cognition lead to lower post-cessation weight concerns in China.

Our findings showed that the perception level of post-cessation weight gain was also quite low; those knowing ‘smoking cessation will affect the weight’, ‘weight gain after smoking cessation belongs to withdrawal symptoms’, ‘weight gain after smoking cessation will occur in some smokers’ and ‘weight gain will be rapid in the first three months after smoking cessation, then slow down and stabilize’ accounted for only 36.07%, 22.37%, 29.6% and 14.95% of the studied population, respectively. Most of the participants thought that smoking cessation had nothing to do with weight gain. According to the ‘peak of ignorance’ in the Dunning-Kruger effect theory^36^, the individuals with low ability or knowledge in a particular domain vastly overestimate their competence, which could partially explain the lower rate of post-cessation weight concerns in this population.

It is worth noting that a positive association between post-cessation weight concerns and intentions to quit smoking were presented in this study, regardless of no adjustment model and multiple variable adjustment model. Our findings were consistent with a study from Sepinwall et al.^10^. Still, it was contrary to most previous studies, because post-cessation weight gain concern was generally viewed as a potential disadvantage factor for quitting smoking in Western countries^19,21,23^. The positive relationship is reasonable, since the general post-cessation weight concern level is low. Those with higher weight concerns in the study population were younger, single, living in urban districts, had a high level of education, started smoking early, with shorter smoking duration but heavy smokers, experienced weight gain in previous quit attempts, and had a higher perception of post-cessation weight gain. They are more likely to pay more attention to health and have higher intentions to quit smoking.

Although weight concerns were not a barrier to reduced intentions to quit smoking currently in male smokers from Chongqing, the largest city of China, weight concerns might affect intentions to quit with the improvement of cognitive level about weight gain after quitting smoking in the future. Large-scale investigations concerning the nationwide status of post-cessation weight concern and its relationship to smoking cessation, are needed in China, the largest market of tobacco in the world. In tobacco control interventions, it is necessary to increase the correct understanding of weight gain after quitting smoking. Proper diet and exercise interventions are needed to alleviate post-cessation weight gain.

### Limitations

The present study is limited by its sites, selected from parks and squares. The sampling method used in this study is convenience sampling, and sample representativeness may not be as good as a sample from a multi-stage random survey. Another limitation is that this study is cross-sectional. Study with prospective data would have provided direct evidence of causality. Furthermore, residual confounding remains in this study. We have no data on participants’ physical health, and there is a certain bias in precluding subjects with severe diseases based solely on self-reports.

## CONCLUSIONS

Due, in part, to the poor perception of post-cessation weight gain, the extent of post-cessation weight concerns was low in male smokers in Chongqing, China. Since a positive correlation between post-cessation weight concerns and intentions to quit smoking has been reported, it appears that weight concerns have not been a significant deterrent to the intention of quit smoking among the participants in this study.

## Data Availability

The data supporting this research are available from the authors on reasonable request.

## References

[1] Zhou M, Wang H, Zeng X, et al. Mortality, morbidity, and risk factors in China and its provinces, 1990-2017: a systematic analysis for the Global Burden of Disease Study 2017. Lancet. 2019;394(10204):1145-1158. doi:10.1016/S0140-6736(19)30427-1 31248666 PMC6891889

[2] Chen Z, Peto R, Zhou M, et al. Contrasting male and female trends in tobacco-attributed mortality in China: evidence from successive nationwide prospective cohort studies. Lancet. 2015;386(10002):1447-1456. doi:10.1016/S0140-6736(15)00340-2 26466050 PMC4691901

[3] Gerber Y, Myers V, Goldbourt U. Smoking reduction at midlife and lifetime mortality risk in men: a prospective cohort study. Am J Epidemiol. 2012;175(10):1006-1012. doi:10.1093/aje/kwr466 22306566

[4] Chang JT, Anic GM, Rostron BL, Tanwar M, Chang CM. Cigarette smoking reduction and health risks: a systematic review and meta-analysis. Nicotine Tob Res. 2021;23(4):635-642. doi:10.1093/ntr/ntaa156 32803250

[5] Parikh NS, Salehi Omran S, Kamel H, Elkind MSV, Willey JZ. Smoking-cessation pharmacotherapy for patients with stroke and TIA: Systematic review. J Clin Neurosci. 2020;78:236-241. doi:10.1016/j.jocn.2020.04.026 32334957 PMC8908464

[6] Duncan MS, Freiberg MS, Greevy RA, Kundu S, Vasan RS, Tindle HA. Association of smoking cessation with subsequent risk of cardiovascular disease. JAMA. 2019;322(7):642-650. doi:10.1001/jama.2019.10298 31429895 PMC6704757

[7] Danan ER, Joseph AM, Sherman SE, et al. Does motivation matter? Analysis of a randomized trial of proactive outreach to VA smokers. J Gen Intern Med. 2016;31(8):878-887. doi:10.1007/s11606-016-3687-1 27071399 PMC4945562

[8] Miskimins K, Kaufmann A, Haaga DAF. Comparative analysis of alternate measures of readiness to quit smoking: stages of change and the contemplation ladder. Subst Abuse Rehabil. 2023;14:167-171. doi:10.2147/SAR.S440691 38089114 PMC10712342

[9] Feng G, Jiang Y, Li Q, et al. Individual-level factors associated with intentions to quit smoking among adult smokers in six cities of China: findings from the ITC China Survey. Tob Control. 2010;19(suppl 2):i6-i11. doi:10.1136/tc.2010.037093 20935198 PMC2976002

[10] Sepinwall D, Borrelli B. Older, medically ill smokers are concerned about weight gain after quitting smoking. Addict Behav. 2004;29(9):1809-1819. doi:10.1016/j.addbeh.2004.04.002 15530723

[11] Chapman S, Wong WL, Smith W. Self-exempting beliefs about smoking and health: differences between smokers and ex-smokers. Am J Public Health. 1993;83(2):215-219. doi:10.2105/ajph.83.2.215 8427326 PMC1694573

[12] Weekley CK, Klesges RC, Reylea G. Smoking as a weight-control strategy and its relationship to smoking status. Addict Behav. 1992;17(3):259-271. doi:10.1016/0306-4603(92)90031-p 1636473

[13] Audrain JE, Klesges RC, Klesges LM. Relationship between obesity and the metabolic effects of smoking in women. Health Psychol. 1995;14(2):116-123. doi:10.1037//0278-6133.14.2.116 7789346

[14] Tian J, Venn A, Otahal P, Gall S. The association between quitting smoking and weight gaIn: a systemic review and meta-analysis of prospective cohort studies. Obes Rev. 2016;17(10):1014. doi:10.1111/obr.12448 27612637

[15] Chinn S, Jarvis D, Melotti R, et al. Smoking cessation, lung function, and weight gaIn: a follow-up study. Lancet. 2005;365(9471):1629-1601. doi:10.1016/S0140-6736(05)66511-7 15885295

[16] Liu G, Hu Y, Zong G, et al. Smoking cessation and weight change in relation to cardiovascular disease incidence and mortality in people with type 2 diabetes: a population-based cohort study. Lancet Diabetes Endocrinol. 2020;8(2):125-133. doi:10.1016/S2213-8587(19)30413-9 31924561 PMC6986932

[17] Hu Y, Zong G, Liu G, et al. Smoking cessation, weight change, type 2 diabetes, and mortality. N Engl J Med. 2018;379(7):623-632. doi:10.1056/NEJMoa1803626 30110591 PMC6165582

[18] Ninomiya Y, Kawasoe S, Kubozono T, et al. Association between weight gain following smoking cessation and development of hypertension in the future. Hypertens Res. 2024;47(5):1167-1174. doi:10.1038/s41440-023-01549-8 38182903

[19] Veldheer S, Yingst J, Foulds G, et al. Once bitten, twice shy: concern about gaining weight after smoking cessation and its association with seeking treatment. Int J Clin Pract. 2014;68(3):388-395. doi:10.1111/ijcp.12332 24471797

[20] Clark MM, Hurt RD, Croghan IT, et al. The prevalence of weight concerns in a smoking abstinence clinical trial. Addict Behav. 2006;31(7):1144-1152. doi:10.1016/j.addbeh.2005.08.011 16137833

[21] Bush T, Levine MD, Deprey M, et al. Prevalence of weight concerns and obesity among smokers calling a quitline. J Smok Cessat. 2008;4(5):74-78. doi:10.1375/jsc.4.2.74 20548969 PMC2884159

[22] Clark MM, Decker PA, Offord KP, et al. Weight concerns among male smokers. Addict Behav. 2004;29(8):1637-1641. doi:10.1016/j.addbeh.2004.02.034 15451131

[23] Jeffery RW, Hennrikus DJ, Lando HA, Murray DM, Liu JW. Reconciling conflicting findings regarding postcessation weight concerns and success in smoking cessation. Health Psychol. 2000;19(3):242-246. doi:10.1037//0278-6133.19.3.242 10868768

[24] Levine MD, Perkins KA, Marcus MD. The characteristics of women smokers concerned about postcessation weight gain. Addict Behav. 2001;26(5):749-756. doi:10.1016/s0306-4603(00)00156-8 11676384

[25] Aubin HJ, Berlin I, Smadja E, West R. Factors associated with higher body mass index, weight concern, and weight gain in a multinational cohort study of smokers intending to quit. Int J Environ Res Public Health. 2009;6(3):943-957. doi:10.3390/ijerph6030943 19440425 PMC2672403

[26] Bush T, Levine MD, Zbikowski S, et al. Weight gain after quitting: attitudes, beliefs and counselling strategies of cessation counsellors. J Smok Cessat. 2008;3(2):124-132. doi:10.1375/jsc.3.2.124 20574550 PMC2889697

[27] Pirie PL, Murray DM, Luepker RV. Gender differences in cigarette smoking and quitting in a cohort of young adults. Am J Public Health. 1991;81(3):324-327. doi:10.2105/ajph.81.3.324 1994740 PMC1405015

[28] Pomerleau CS, Zucker AN, Namenek Brouwer RJ, Pomerleau OF, Stewart AJ. Race differences in weight concerns among women smokers: results from two independent samples. Addict Behav. 2001;26(5):651-663. doi:10.1016/s0306-4603(00)00148-9 11676376

[29] King TK, Matacin M, White KS, Marcus BH. A prospective examination of body image and smoking cessation in women. Body Image. 2005;2(1):19-28. doi:10.1016/j.bodyim.2005.01.003 18089171

[30] Zhang M, Yang L, Wang L, et al. Trends in smoking prevalence in urban and rural China, 2007 to 2018: Findings from 5 consecutive nationally representative cross-sectional surveys. PLoS Med. 2022;19(8):e1004064. doi:10.1371/journal.pmed.1004064 36006870 PMC9409540

[31] Borrelli B, Mermelstein R. The role of weight concern and self-efficacy in smoking cessation and weight gain among smokers in a clinic-based cessation program. Addict Behav. 1998;23(5):609-622. doi:10.1016/s0306-4603(98)00014-8 9768298

[32] Heatherton TF, Kozlowski LT, Frecker RC, Fagerström KO. The Fagerström test for nicotine dependence: a revision of the Fagerström Tolerance Questionnaire. Br J Addict. 1991;86(9):1119-1127. doi:10.1111/j.1360-0443.1991.tb01879.x 1932883

[33] Collins BN, Nair U, Hovell MF, Audrain-McGovern J. Smoking-related weight concerns among underserved, black maternal smokers. Am J Health Behav. 2009;33(6):699-709. doi:10.5993/ajhb.33.6.7 19320618 PMC3715964

[34] Pollak KI, Namenek Brouwer RJ, Lyna P, Taiwo B, McBride CM. Weight and smoking cessation among low-income African Americans. Am J Prev Med. 2003;25(2):136-139. doi:10.1016/s0749-3797(03)00118-1 12880881

[35] Meyers AW, Klesges RC, Winders SE, Ward KD, Peterson BA, Eck LH. Are weight concerns predictive of smoking cessation? A prospective analysis. J Consult Clin Psychol. 1997;65(3):448-452. doi:10.1037//0022-006x.65.3.448 9170768

[36] Kruger J, Dunning D. Unskilled and unaware of it: how difficulties in recognizing one’s own incompetence lead to inflated self-assessments. J Pers Soc Psychol. 1999;77(6):1121-1134. doi:10.1037//0022-3514.77.6.1121 10626367

